# The Protean Toxicities of Lead: New Chapters in a Familiar Story

**DOI:** 10.3390/ijerph8072593

**Published:** 2011-06-27

**Authors:** David C. Bellinger

**Affiliations:** 1 Children’s Hospital Boston, Farley Basement Box 127, 300 Longwood Avenue, Boston, MA 02115, USA; E-Mail: david.bellinger@childrens.harvard.edu; Tel.: +1-617-355-6565; Fax: +1-617-730-0618; 2 Harvard Medical School, Harvard University, 25 Shattuck Street, Boston, MA 02115, USA; 3 Harvard School of Public Health, Harvard University, 677 Huntington Avenue, Boston, MA 02115, USA

**Keywords:** lead, epidemiology, adults, children

## Abstract

Many times in the history of lead toxicology the view that “the problem” has been solved and is no longer a major health concern has prevailed, only to have further research demonstrate the prematurity of this judgment. In the last decade, an extraordinary amount of new research on lead has illustrated, all too clearly, that “the problem” has not disappeared, and that, in fact, it has dimensions never before considered. Recent risk assessments have concluded that research has yet to identify a threshold level below which lead can be considered “safe.” Although children’s intelligence has traditionally been considered to be the most sensitive endpoint, and used as the basis for risk assessment and standard setting, increased lead exposure has been associated with a wide variety of other morbidities both in children and adults, in some cases at biomarker levels comparable to those associated with IQ deficits in children. In adults, these endpoints include all-cause mortality and dysfunctions in the renal, cardiovascular, reproductive, central nervous systems. In children, IQ deficits are observed at blood lead levels well below 10 μg/dL, and the dose-effect relationship appears to be supra-linear. Other health endpoints associated with greater early-life lead exposure in children include ADHD, conduct disorder, aggression and delinquency, impaired dental health, and delayed sexual maturation. Studies employing neuroimaging modalities such as volumetric, diffusion tensor, and functional MRI are providing insights into the neural bases of the cognitive impairments associated with greater lead exposure.

## 1. Introduction

Many times in the history of lead toxicology the view that “the problem” has been solved and is no longer a major health concern has prevailed, only to have further research demonstrate the prematurity of this judgment. In the last decade, an extraordinary amount of new research on lead has illustrated that “the problem” has clearly not disappeared, and that, furthermore, it has dimensions never before considered. This review broadly surveys the epidemiologic literature published in the last decade on the health effects of lead exposure, both in adults and children, focusing on the lowest observed adverse effect levels, the shapes of the pertinent dose-effect/dose-response relationships, and susceptible subgroups.

## 2. Mortality

Recent prospective cohort studies conducted in cohorts drawn from the general population provide reasonably consistent evidence, in both men and women, that greater blood lead levels are associated with higher all-cause mortality and that deaths from cardiovascular diseases are largely responsible for the associations. This association is apparent in the range of blood lead levels below 10 μg/dL.

Early studies using data from the National Health and Nutrition Examination Survey (NHANES) suggested that a baseline blood lead level in the range of 20 to 29 μg/dL was significantly associated with all-cause mortality, using individuals with a baseline blood lead level below 10 as the reference group [1]. Individuals with a baseline blood lead level of 10–19 μg/dL were also at increased risk, but not significantly so.

As population blood lead levels have fallen, it has become been possible to evaluate associations in the range below 10 μg/dL. Menke *et al.* [2] followed up, after 12 years, 13,946 participants in the NHANES III survey (1988–1994) who had a baseline blood lead level less than 10 μg/dL (mean 2.6 μg/dL). The causes of death considered were cardiovascular disease, myocardial infarction, stroke, cancer and lung cancer. Cox proportional hazard regression was used to estimate hazard ratios (HRs) for individuals in baseline blood lead tertiles, adjusting for age, race, sex, diabetes mellitus, body mass index, smoking, alcohol consumption, physical activity, income, C-reactive protein, total cholesterol, education, urban residence, postmenopausal status, hypertension and kidney function (glomerular filtration rate [GFR] <60 mL/min/1.73 m^3^). Comparing individuals in the highest tertile to those in the lowest tertile, the adjusted HR for all-cause mortality was 1.25 (95% CI 1.0–1.5, P for trend = 0.002). The associations with baseline blood lead level were also significant for cardiovascular deaths (HR 1.6, 95% CI 1.1–2.2), myocardial infarction (HR 1.9, 95% CI 1.0–3.4) and stroke (HR 2.5, 95% CI 1.2–5.3). Spline regressions, used to describe the shapes of the relationships, suggested that the increase in mortality was evident when blood lead level exceeded 2 μg/dL.

In another analysis of NHANES III (N = 9,757), Schober *et al.* [3] compared mortality risk of individuals with a baseline blood lead level below 5 μg/dL and individuals with a level of 5–9 μg/dL. The latter group had a significantly increased adjusted risk of all-cause mortality (HR 1.24, 95% CI 1.05–1.48), as did individuals with a baseline blood lead of 10 μg/dL or higher (HR 1.59, 95% CI 1.28–1.98). The HRs were similar in these two exposure strata for deaths from cardiovascular disease or cancer.

The associations between lead biomarkers and total and cause-specific mortality were also evaluated, over a follow-up interval of nine years, in another cohort, 868 men (mean age 67 years) enrolled in the United States Veterans Administration Normative Aging Study (NAS) [4]. Blood lead level at baseline, which averaged 5.6 μg/dL(SD 3.4), was not associated with mortality, but lead concentration in patella, measured by K-line X-ray fluorescence, was significantly associated with all-cause and with cardiovascular and ischemic heart disease deaths, adjusting for age, smoking and education. The HR for men in the highest tertile of patella lead, compared with men in the lowest tertile, was 2.5 (95% CI 1.2–5.4). Adjustment for additional covariates, including hypertension, race, alcohol use, physical activity, body mass index, high-density lipoprotein, cholesterol and diabetes mellitus, did not alter the results appreciably. Analyses that explored the functional forms of the associations suggested linear dose-response relationships. For all three endpoints, the HRs associated with tibia lead concentration were not significant.

Khalil *et al.* [5] conducted a 12-year follow-up study of 533 women, ages 65–87 at baseline. The mean blood lead level at baseline was 5.3 μg/dL (SD 2.3) (range 1–21). For all-cause mortality, the HR for women with a blood lead level of >8 μg/dL was 1.6 (95% CI 1.0–2.5, P = 0.04), compared to women with a level <8 μg/dL. For deaths from cardiovascular diseases, the HR was 3.1 (95% CI 1.2–7.7, P = 0.02). Blood lead level was not significantly associated with stroke, cancer, or non-cardiovascular deaths.

## 3. Cancer

In 2006, IARC [6] reviewed studies of the carcinogenicity of inorganic lead, considering studies of both occupational and environmental (*i.e.*, general population) exposures. IARC concluded that although the evidence for carcinogenicity is sufficient in animals, there is, “limited evidence” in humans for the carcinogenicity of inorganic lead and that inorganic lead compounds are “probably carcinogenic” to humans (group 2A).

Since the IARC evaluation, the results of additional studies published do not suggest a need to revise this conclusion. In a study using the data on 13,946 participants in NHANES III (1988–1994), Menke *et al.* [2] evaluated the association between blood lead level (mean 2.6 μg/dL) and overall cancer mortality and mortality from lung cancer. In analyses adjusting for age, race/ethnicity, sex, diabetes mellitus, body mass index, smoking, alcohol consumption, physical activity, income, C-reactive protein, total cholesterol, education, residence, postmenopausal status, hypertension and kidney function, the HR for individuals in tertile 2 of blood lead level (1.94–3.62 μg/dL) was 0.72 (95% CI 0.46–1.12). The HR for individuals in tertile 3 of blood lead level (>−3.63 μg/dL) was 1.10 (95% CI 0.82–1.47). The P for trend was 0.10. The HRs for lung cancer in the tertiles 2 and 3 were 0.70 (95% CI 0.34–1.42) and 0.79 (95% CI 0.40–1.58), respectively.

Among 868 men participating in the United States Veterans Administration Normative Aging Study (NAS), Weisskopf *et al.* [4] found that neither baseline blood lead level nor patella lead level was significantly associated with cancer mortality. For baseline blood lead level, adjusting for age, smoking and education, the HR for individuals in tertile 2 (4–6 μg/dL) was 1.03 (95% CI 0.42–2.55), and the HR for individuals in tertile 3 (>6 μg/dL) was 0.53 (95% CI 0.20–1.39). For patella lead level, the HR for individuals in tertile 2 (22–35 μg/g) was 0.82 (95% CI 0.26–2.59), and the HR for individuals in tertile 3 (>35 μg/g) was 0.32 (95% CI 0.08–1.35).

In a case-control study of primary smelter workers, arsenic exposure, but not lead exposure, was a risk factor for lung cancer in 141 cases and age-matched controls [7].

Some recent studies suggest associations that warrant future consideration. Alatise and Schrauzer [8] reported that women newly diagnosed with infiltrating ductal carcinoma of the breast had higher levels of blood and hair lead than controls and that hair lead level correlated significantly with tumor volume. In a study of 362 patients with brain tumors (glioma or meningioma) and 494 controls, gene-environment interactions were found [9]. Specifically, cumulative lead exposure as a main effect, estimated on the basis of job history, was not associated with glioblastoma multiforme and meningioma, but polymorphisms in the RAC2 and GPX1 genes (for glioblastoma multiforme) and the GPX1 gene and the XDH gene (for meningioma) were observed to modify the association.

## 4. Renal

Lead has long been known to be a renal toxicant. Some recent studies focused on patients with clinical kidney disease, with some evaluating whether greater lead exposure is associated with the loss of kidney function. A large study of incident chronic kidney disease (CKD) cases among lead workers in Sweden (926 cases, 998 controls) failed to find an increased risk of CKD or faster rate of decline in GFR over a 7–9 year follow-up interval [10]. Air lead levels, but not lead biomarker measurements, were available, however.

Lin and colleagues reported a series of studies on patients with CKD, evaluating whether the rate of decline in kidney function over time differed depending on lead burden. Some studies were observational. Yu *et al.* [11] followed 121 patients for four years, classifying their baseline ethylenediaminetetraacetic acid (EDTA)-chelatable lead as “low” (urinary lead level below 80 μg/72 h urine collection following a provocative chelation dose) or “high” (urinary lead level 80 to 600 μg/72 h). Significantly more patients with high compared with low baseline lead burdens experienced a doubling of serum creatinine level or required hemodialysis (P = 0.001). Each μg/dL increase in baseline blood lead level, which averaged 4.9 μg/dL in the high-chelatable lead group and 3.4 μg/dL in the low-chelatable lead group, was associated with a reduction of 4.0 mL/min per 1.73 m^3^ in GFR over the period of observation. Other studies by this group involved random assignment of patients with chronic kidney disease to receive therapeutic chelation, with decline in kidney function as the primary end-point. In a study involving 64 patients with baseline chelatable lead levels of 80 to 600 μg/72 h, patients randomized to active treatment received EDTA for up to three months, with additional treatment as needed [12]. The mean baseline blood lead levels of the chelation and placebo groups were 6.1 and 5.9 μg/dL, respectively. At the end of two years, the mean estimated GFR had increased by 2.1 mL/min per 1.73 m^3^ in the chelated group and declined by 6.0 mL/min per 1.73 m^3^ in the placebo group (P < 0.01). A subsequent study involved 108 patients with CKD, chelatable lead levels of 20 to 80 μg/72 h, and baseline blood lead levels of 1.2–4.6 μg/dL [13]. The mean change in GFR was 6.6 mL/min per 1.73 m^3^ in the chelated group and −4.6 in the placebo group (P < 0.001). This group conducted a study similar in design on 87 patients with type II diabetes and diabetic nephropathy, baseline chelatable lead levels between 30 and 373 μg/72 h and a mean blood lead level of 6.5 μg/dL (range 1.6–19.1 μg/dL) [14]. In the 12-month observation period following random assignment of patients to chelation or placebo, the rate of decline in GFR was 5.0 (SD 5.7) mL/min per year per 1.73 m^3^ in the chelation group and 11.8 (SD 7.0) mL/min per year per 1.73 m^3^ in the placebo group. In these patients with diabetes, higher baseline blood and chelatable lead levels were both significantly associated with increased risk of progressive nephropathy.

A case-control study compared the blood and tibia lead levels of 55 African Americans with end-stage renal disease receiving chronic hemodialysis treatment with those of 53 age- and sex-matched controls [15]. The cause of end-stage renal disease was hypertension for 40% of the cases, diabetes for 36%, glomerulosclerosis for 6% and unknown for 18%. The mean blood lead level was significantly higher among cases (6 *versus* 3 μg/dL, P < 0.001), with 67% of cases (compared with 6% of controls) having a level of 5–9 μg/dL, and 15% (compared with no controls) having a level ≥10 μg/dL. The tibia lead levels of cases were somewhat higher than those of controls, but the difference was not significant. The authors suggested that this finding, along with the fact that blood and tibia lead levels were more highly correlated for cases than for controls, might indicate greater bone turnover in the cases, resulting in higher blood lead levels.

Recently, studies have evaluated the association between increased lead exposure and subtle renal impairment in the general population. Using adult (≥20 years old) participants in NHANES III (N = 15,211), Muntner *et al.* [16] considered two indices of renal function: serum creatinine (elevation defined as greater than the 99th percentile for race/sex) and chronic kidney disease (GFR < 60 mL/min per 1.73 m^3^). Models were adjusted for age, sex, systolic blood pressure, diabetes mellitus, current smoking, history of cardiovascular disease, body mass index, alcohol consumption, household income, marital status and health insurance. Significant associations between blood lead level and kidney dysfunction were found among individuals with hypertension (N = 4,813), but not among those free of hypertension (N = 10,398). Among those with hypertension, after adjustment for covariates, individuals in the highest quartile of blood lead level (6.0–56.0 μg/dL), compared to individuals in the lowest quartile, were 2.4 (95% CI 1.5–4.0) times more likely than individuals in the lowest quartile (0.7–2.4 μg.dL) to have an elevated serum creatinine level. Similarly, these individuals were 2.6 (95% CI 1.5–4.5) times more likely than the individuals in the lowest quartile to have chronic kidney disease. For both outcomes, the adjusted ORs were also significant for individuals in quartiles 2 (2.5–3.8 μg/dL) and 3 (3.9–5.9 μg/dL), with the trend across quartiles significant (P < 0.001). Muntner *et al.* [17] reported similar associations using data from NHANES 1999–2002 (N = 9,961). Adjusting for the same set of covariates plus race/ethnicity, individuals in the highest quartile of blood lead level (≥2.47 μg/dL) were 2.7 (95% CI 1.5–5.0) times more likely than individuals in quartile 1 of blood lead level (<1.1 μg/dL) to have chronic kidney disease (GFR <60 mL/min per 1.73 m^3^).

In a sample of adults from Taiwan, China (n = 1,565), Lai *et al.* [18] evaluated the associations between blood lead level and elevated serum creatinine level (>1.2 mg/dL) and elevated serum uric acid level (>7 mg/dL in males and >6 mg/dL in females). Adjusting for age, sex, occupation, education, marital status, smoking, alcohol, betel nut chewing, hypertension and lipid levels, the ORs for individuals with blood lead levels in the highest tertile (>7.5 μg/dL; 0.8% ≥10 μg/dL) were 1.9 (95% CI 1.2–3.1) for elevated serum creatinine level and 2.7 (95% CI 1.6–4.5) for hyperuricaemia (both P < 0.01).

One study investigated the association between lead exposure and change over time in renal function. In the NAS cohort, Tsaih *et al.* [19] found that the lead-related decline in renal function over a 6-year follow-up interval, specifically the rate of rise in serum creatinine level, was greater in individuals who, at baseline, had diabetes. For example, for an increase in tibia lead level corresponding to the difference between the midpoints of the lowest and highest quartiles (9–34 μg/g), the rate of increase was 17.6-fold greater in diabetics than in non-diabetics (1.08 mg/dL/10 years *vs.* 0.062 mg/dL/10 years; p < 0.01).

Relatively little information is available on lead exposure and renal function in children. In a sample of 769 healthy 12–20 year olds in NHANES III, Fadrowski *et al.* [20] evaluated the association between blood lead level and GFR, estimated on the basis of both serum cystatin C level and serum creatinine level. Serum cystatin C appears to be less dependent than creatinine on age, sex, height and muscle mass and so might be a better marker of kidney function. The median blood lead level was 1.5 μg/dL (interquartile range 0.7–2.9 μg/dL). Models were adjusted for age, sex, race/ethnicity, urban/rural, tobacco smoke exposure, annual household income and educational level of family reference person. Adolescents with a blood lead level in the highest quartile (≥3 μg/dL) had a 6.6 mL/min per 1.73 m^3^ lower cystatin C-estimated GFR (95% CI −0.7 to −12.6 mL/min per 1.73 m^3^) compared with those in the first quartile (<1 μg/dL). The trend was significant trend (P = 0.009), and restricted quadratic spline analyses showed neither a departure from linearity nor a threshold. The associations were qualitatively similar but weaker when creatinine level was used to estimate GFR, suggesting that studies that rely on creatinine-based estimates of kidney function might underestimate the association between GFR and blood lead level. The use of a cross-sectional design leaves open the possibility of reverse causation (*i.e.*, kidney disease causes decreased excretion of lead), but this seems unlikely, as at least some prospective studies have shown that baseline blood lead level is associated with subsequent decline in kidney function [21], particularly among participants with diabetes or hypertension [19].

The few data available on lead exposure and renal function in even younger children suggest that higher blood lead levels are associated with increased GFR (as estimated by serum creatinine or cystatin C levels), suggesting a paradoxical effect that, perhaps, reflects a hyperfiltration phenomenon [22,23].

Several factors have been reported to modify the association between blood lead level and kidney function, although the evidence is inconsistent. These include certain genetic polymorphisms, including ALAD, the vitamin D receptor and nitric oxide synthase [24–26]. Among adults who participated in NHANES 1999–2006 (N = 14,778), higher cadmium exposure resulted in more striking positive associations between blood lead level and renal dysfunction [27]. Adjusting for survey year, age, sex, race/ethnicity, body mass index, education, smoking, cotinine, alcohol consumption, hypertension, diabetes mellitus and menopausal status, individuals with both blood lead and blood cadmium levels in the highest quartile >0.6 μg/L for cadmium, >2.4 μg/dL for lead) were 2.3 (95% CI 1.7–3.2) times more likely than individuals in the lowest quartiles for both lead and cadmium to have albuminuria and 2.0 (95% CI 1.3–3.1) times more likely to have a reduced GFR (estimated based on serum creatinine). If blood cadmium level was not considered, the individuals in the highest quartile for lead were 1.2 (95% CI 1.0–1.5) times more likely to have albuminuria and 1.6 (95% CI 1.2–2.1) times more likely to have a reduced GFR. Individuals in the highest quartiles of both metals were 4.1 (95% CI 1.6–10.7) times more likely to have both indicators of kidney dysfunction.

## 5. Cardiovascular

Like renal impairment, hypertension has long been recognized as a consequence of occupational exposure to lead, raising the question of whether a similar, perhaps more modest, association is evident between lead burden and blood pressure, as well as other indicators of cardiovascular function, at the lower lead exposures experienced by the general population. Most reviews (e.g., [28]) have concluded that there is a positive relationship between blood lead level and blood pressure, although the association based on the combined evidence is not always statistically significant, however. In a meta-analysis published in 2002, Nawrot [29] found that the change in systolic pressure associated with a doubling in blood lead level was 1.0 mmHg (95% CI 0.5–1.4), and the change in diastolic pressure for a doubling of blood lead level was 0.6 mmHg (95% CI 0.4–0.8).

Among studies conducted subsequent to those included in the Nawrot *et al.* meta-analysis, some suggest that the association between lead exposure and blood pressure varies across sociodemographic strata. Limiting analyses of NHANES III data to women aged 40–59 years (N = 2,165), Nash *et al.* [30] reported that, compared with women with blood lead levels in the lowest quartile (0.5–1.6 μg/dL), women in the highest quartile (4–31 μg/dL) were 3.4 (95% CI 1.3–8.7 ) times as likely to have diastolic hypertension (>90 mmHg) and 1.5 (95% CI 0.7–3.2) times more likely to have systolic hypertension (>140 mmHg). The associations were strongest for postmenopausal women. In analyses of NHANES III data stratifying by race, Vupputuri *et al.* [31] found significant adjusted associations between blood lead level and blood pressure in black males and females. Each 3.3 μg/dL increase was associated with a 0.82 mmHg increase in systolic blood pressure in black males (95% CI 0.19–1.44) and a 1.55 mmHg increase in systolic pressure in black females (95% CI 0.47–2.64). No associations were found in white males or females, however.

The associations between lead and blood pressure might also differ depending on the exposure biomarker used. Park *et al.* [32] used data on the NAS cohort to develop a model for predicting bone lead level from blood lead level in order to reanalyse the association between lead and hypertension in NHANES III. The association was stronger if estimated bone lead level was used in place of blood lead level, suggesting that use of a biomarker of shorter-term exposure, such as blood lead, might produce an underestimate of the association.

The mechanisms suggested for the association between lead exposure and blood pressure include lead-related impairments in renal function, oxidative stress, effects on the rennin-angiotensin system and suppression of nitric oxide. Two studies have suggested another potential mechanism, a lead-related increase in homocysteine level. One study was in a random sample of 1,140 50- to 70-year-olds (the Baltimore Memory Study) [33], and the other was a smaller cross-sectional study in occupationally exposed workers [34].

As noted previously, deaths from cardiovascular disease are usually found to account for much of the association between lead and overall mortality [1,3]. The evidence regarding lead and clinical cardiovascular end-points in the general population is mixed, however. In cross-sectional analyses of NHANES 1999–2002, an association was reported between concurrent blood lead level and the risk of peripheral artery disease [17,35]. Other studies have shown a non-significant elevation in risk of stroke. In a study of heart rate variability (N = 331), comparing blood lead levels <1.39 μg/dL with those >3.45 μg/dL), Jhun *et al.* [36] reported inverse associations between lead and low-frequency, high-frequency and total power spectrum. These associations were not significant in adjusted analyses, however. In the NAS cohort of adult men (N = 413), Park *et al.* [37] also did not find significant adjusted associations between higher tibia or patella lead levels and indices of heart rate variability, although they did find, in men with metabolic syndrome, significant associations between patella lead level and heart rate variability (higher low frequency power and the ratio of low to high frequency power). No associations were found for tibia lead level, however. The authors interpreted these findings as evidence that oxidative stress induced by lead exposure was responsible for autonomic dysfunction of the cardiovascular system. Results consistent with this hypothesis were reported in additional analyses of the same cohort (N = 593), showing that tibia lead level was associated with pulse pressure (the difference between systolic and diastolic pressures), an index of arterial stiffening, but not between blood lead level and pulse pressure [38]. One mechanism of arterial stiffening is thought to be vascular oxidative stress. Men with tibia lead levels greater than the median value (19.0 μg/g) had pulse pressures that were 4.2 mmHg higher (95% CI 1.9–6.5 mmHg), compared with men with tibia lead levels below the median, adjusting for age, race, diabetes, family history of hypertension, education, waist circumference, alcohol intake, smoking, height, heart rate, fasting glucose and ratio of total cholesterol to high density lipoprotein. Patella lead levels were also measured, but results were not reported. Another study in this cohort focusing on electrocardiographic conduction changes over an 8 year follow-up interval showed that men in the highest tertile of tibia lead level had a 7.9 ms increase (95% CI 1.4–14.4) in QTc interval and a 5.9 ms increase (95% CI 1.7–10.2) in QRSc [39]. The more highly exposed men also had increased odds of QT prolongation (QTc ≥ 440 ms, OR = 2.5, 95% CI 1.2–5.2) and JT prolongation (JTc ≥ 360 ms; OR = 2.5, 95% CI: 0.9–6.9). No associations were seen between these endpoints and blood lead level.

Limited data are available on the association between lead exposure and blood pressure in younger age groups. Gerr *et al.* [40] reported significant adjusted associations between higher tibia lead levels and higher systolic and diastolic blood pressures in young adults. Subjects in the highest quartile (>10 μg/g) had systolic pressures 4.3 mmHg higher than those in the lowest quartile (<1 μg/g) and diastolic pressures 2.8 mmHg higher. Although the current blood lead levels were low for subjects in all tibia lead quartiles and were unrelated to either systolic or diastolic blood pressure, the subjects with tibia lead levels in the highest quartile were estimated to have had a mean childhood blood lead level of 65 μg/dL. In the Kosovo prospective lead study [41], a 10 μg/dL increase in blood lead was associated with small increases in systolic pressure (0.5 mmHg, 95% CI −0.2 to 1.3) and diastolic blood pressure (0.4 mmHg, 95% CI −0.1 to 0.9). In a cohort of 12–33 month old children (N = 780), no association was found between blood lead level and blood pressure, but the interpretation of this study is complicated by the fact that the children were enrolled in the Treatment of Lead-Exposed Children Study [42], a randomized clinical trial in which oral succimer was administered to children with a baseline blood lead level of 20–44 μg/dL.

A series of studies in 9–11 year olds with blood lead levels closer to general population levels suggest that early lead exposure (blood lead level measured at a mean of 2.6 years: mean 4.0 μg/dL, range 1.5–13.0) mediates the association between lower family socioeconomic status and greater salivary cortisol response to acute stress. Additional studies of this cohort suggested that that early lead exposure produces cardiac dysregulation, expressed as altered patterns of sympathetic and parasympathetic activation in response to such stress [43–47].

## 6. Reproduction

Lead at high dose impairs reproductive outcomes, and a variety of endpoints pertaining to have been evaluated in relation to maternal and paternal exposures. A recent review concluded that fertility is reduced in couples during periods in which an occupationally-exposed male has a blood lead level >40 μg/dL or a blood lead level in the range of 25 μg/dL for several years [48]. The reduced fertility is manifested as fewer live births, reduced likelihood of conception or increased time to pregnancy. Although the evidence on lead and spontaneous abortion is limited, in one well-designed study of 668 women in Mexico [49], the risk was doubled (OR = 2.3) at maternal blood lead levels of 5–9 μg/dL and was 5-fold higher (OR = 5.4) at a maternal blood lead level of 10–14 μg/dL.

High-dose lead exposure has long been recognized as a risk factor for eclampsia [50], and a recent case-control study suggested that risk is increased even among women with blood lead levels <20 μg/dL [51]. Several studies have investigated the link between lead exposure and pregnancy hypertension, with two case-control studies suggesting that the risk is increased at blood lead levels <10 μg/dL [52,53]. A prospective cohort study [54] found that the lead concentration in the calcaneus, but not in the tibia or in blood, was significantly associated with third-trimester hypertension. Increased lead exposure has also been linked to blood pressure in women during labor and delivery. Among 285 women with a mean blood lead level of 0.66 μg/dL, those with a blood lead level in the highest quartile had a systolic blood pressure at admission that was 6.9 mmHg higher (95% CI 1.5–12.2) and a diastolic blood pressure at admission that was 4.4 mmHg higher (95% CI 0.2–15.5) than women in the lowest quartile of blood lead level, adjusting for age, race, median household income, parity, smoking, pre-pregnancy body mass index, and anemia [55].

In a study conducted in Kosovo [56], the OR for proteinuria was 4.5 (95% CI 1.5–13.6) for women in the highest decile of pregnancy blood lead level (>40 μg/dL), although the OR rose above unity for women with a blood lead level greater than 5.8 μg/dL.

The evidence that increased paternal or maternal lead exposure is associated with the risk of a congenital malformation in offspring is inconsistent. In some studies reporting an association, exposure classification was based solely on job title rather than a lead biomarker, precluding estimation of the critical dose. An increased risk of neural tube defects has been reported in an ecologic study of women residing in an area with high lead levels in water [57]. A study that used data from a regional birth defect surveillance program in the US found that men who were considered presumed, on the basis of self-report, industrial hygiene assessment or job exposure matrix, to have been exposed to lead in the 3-month period prior to conception through the first trimester had an OR of 1.83 (95% CI 1.00–3.42) of having a child with a specific congenital cardiac lesion, total anomalous pulmonary venous return [58]. For maternal exposure during this interval, the OR was 1.57 (95% CI 0.64–3.47).

Considerable evidence supports the hypothesis that adverse effects on fetal growth occur within the range of general population exposures. In Mexican women, women with a tibia lead level in the highest quintile at one month postpartum were 1.79 (95% CI 1.10–3.22) times more likely than infants in the other four quintiles to have a below average birth length [59]. In the same cohort, infants of mothers with higher patella lead levels at one month postpartum had smaller head circumference [59], lower weight at one month of age, and less weight gain between birth and one month [60]. Similar relationships between cord blood lead level (mean 3.9 μg/dL, SD 3.6) and birth weight and length were reported in a Brazilian study [61]. In a study of 262 pregnancies in California [62], women with a blood lead level >10 μg/dL during pregnancy were at increased risk of delivering an infant that was preterm (OR 3.2, 95% CI 1.2–7.4) or small for gestational age (OR 4.2, 95% CI 1.3–13.9). Second-trimester blood lead level was a particularly strong predictor of length of gestation (−1.0 days/μg/dL >10 μg/dL). Using data from the New York State Heavy Metals Registry, Zhu *et al.* [63] found that among 43,288 women with blood lead levels <10 μg/dL, birth weight bore an inverse supra-linear relationship to blood lead level during pregnancy. Neither the risk of preterm birth nor small-for-gestational age was significantly elevated, however.

The U.S. Centers for Disease Control and Prevention [64] recently issued revised guidelines for the management of lead exposure during pregnancy, suggesting that a blood lead level >5 μg/dL should trigger follow-up activities and interventions.

## 7. Nervous System

### 7.1. Nerve Conduction Velocity

A recent meta-analysis evaluated the association between blood lead level and peripheral nerve conduction velocities, latencies and amplitudes in adults [65]. Forty-nine studies included 2,825 individuals exposed primarily as a result of occupation, and 1629 controls. The nerves assessed included the median, ulnar and radial nerves in the arm and the deep and superficial peroneal, posterior tibial, aural and fibular nerves in the leg. In mixed models, the slopes of the relationships were generally negative for velocities, positive for latencies, and flat for amplitudes. The lowest blood lead level at which relationships were observed ranged from 33.0 μg/dL for conduction velocity of the median sensory nerve to 64.0 μg/dL for distal motor latency of the median nerve. Because the participants were occupationally-exposed, however, the current blood lead level might be a poor index of the dose required to produce the nerve dysfunctions observed.

### 7.2. Postural Balance

The association between blood lead level and postural sway, measured using the Neuromotor Test System (CATSYS), was evaluated in a cohort of 181 workers (121 lead-exposed, 60 controls) [66]. Analyses were adjusted for age, height, smoking and alcohol use. Most indices of postural sway were significantly worse in the workers and associated with blood lead level. Benchmark dose (BMD) modelling of the several indices of sway produced lower bounds on the BMD in the range of 12–17 μg/dL.

### 7.3. Essential Tremor (ET)

In a case-control study, the mean blood lead level of 100 patients with ET (3.3 μg/dL [SD 2.4]) was significantly higher than the mean blood lead level of 143 controls (2.6 μg/dL [SD 1.6]) [67]. In a logistic regression analysis adjusting for age and current cigarette smoking, the increase in risk of ET per unit increase in blood lead level was 1.19 (95% CI 1.03–1.57, P = 0.02). The adjusted OR was somewhat greater when patients with a family history of ET were excluded (OR 1.38, 95% CI 1.15–1.64, P = 0.001). The same authors reported that total tremor scores were significantly greater in participants who had high blood levels of both lead and harmane, a β-carboline alkaloid known to produce tremor [68]. In a separate cohort of 105 ET cases and 105 controls [69], the mean blood lead level of cases was 3.2 μg/dL (SD 1.9) and 1.6 μg/dL (SD 0.8) in controls. Adjusting for age, sex, education, cigarette smoking, cigarette pack-years and alcohol use, the OR was 4.19 (95% CI 2.59–6.78, P < 0.001). In addition, the correlation between tremor severity and blood lead level among ET cases was 0.48 (P < 0.001).

Neither of these studies involved incident cases of ET, and the cross-sectional design used in each makes it uncertain whether the higher blood lead levels of the cases preceded or followed the diagnosis of ET. Even if elevated lead exposure preceded the diagnosis and the role of lead is causal, these studies are of limited use in identifying the critical dose. Because the mean age of participants was greater than 50 years in both studies, their blood lead levels might have been considerably higher in the past, when the damage leading to ET is likely to have occurred.

### 7.4. Amyotrophic Lateral Sclerosis (ALS)

Past lead exposure has been associated with the risk of ALS in case-control studies [70–72] and a case report [73]. In Kamel *et al.* [70], information on lead exposure was obtained for 109 cases and 256 community controls frequency matched to cases on age, sex and residence. Bone and blood lead levels were measured in 107 cases and 41 controls. Individuals who self-reported occupational exposure to lead were 1.9 (95% CI 1.1–3.3) times more likely to have ALS. The risk of ALS increased with increasing patella lead level (OR 3.6, 95% CI 0.6–20.6, for each μg/g increase), tibia lead level (OR 2.3, 95% CI 0.4–14.5, for each μg/g increase), and blood lead level (OR 1.9, 95% CI 1.4–2.6, for each μg/dL increase). A follow-up study found a weak association, using Cox proportional hazard analysis, between longer survival and higher baseline blood lead level (HR 0.9, 95% CI 0.8–1.0), baseline patella lead level (HR 0.5, 95% CI 0.2–1.0) and baseline tibia lead level (HR 0.3, 95% CI 0.1–0.7) [71]. In another study (184 cases, 194 controls), a doubling of blood lead level was associated with a 1.9-fold (95% CI 1.3–2.7) increase in risk of ALS, adjusting for age and bone resorption rate (C-terminal telopeptides of type 1 collagen) [72]. Additional adjustment for an index of bone formation (procollagen type 1 amino-terminal peptide) did not affect the results.

### 7.5. Adult Cognitive Function

Many of the studies evaluating the association between lead exposure and adult cognitive function have relied on bone lead level as the exposure biomarker. Beyond the lack of widespread capacity to measure bone lead levels, it is difficult to integrate the results of studies that used these two biomarkers. The correlation between bone lead level and a concurrent blood lead level is often poor because of the very different exposure averaging times that these two biomarkers capture. In a study of 50- to 70-year-olds with primarily environmental exposures to lead, the correlation between bone and blood lead levels was only 0.12 [74]. The correlation can be much higher, however, in individuals who, in the past, had substantial occupational exposures to lead, for whom bone stores contributed to blood lead in later life, after occupational exposure ended [75].

Seeber, *et al.* [76] reviewed two meta-analyses of 24 studies of cognitive test scores in workers occupationally exposed to lead, concluding that although the evidence is not entirely consistent across studies, deficits in various domains are present at blood lead levels of 37 to 52 μg/dL. Khalil *et al.* [77] administered a battery of tests to 83 battery plant workers and 51 controls who had previously been part of a cohort of 469 individuals administered the same battery 22 years earlier. Current mean blood lead level was 12 μg/dL for the workers and 3 μg/dL for the controls. Tibia lead level was associated with lower scores, both cross-sectionally and longitudinally, predicting the decline in performance over the follow-up interval in the workers, but not in the controls, adjusting for baseline scores, age, education, years of employment and lifestyle factors. The domains most strongly related to cumulative lead exposure were spatial ability, executive functions and learning/memory. Schwartz *et al.* [78] also reported that bone lead level predicts the magnitude of the decline over time in the scores of lead workers.

Similar findings have been reported in cohorts drawn from the general population. In the Baltimore Memory Study, a longitudinal study of urban adults of diverse ethnicity, Bandeen-Roche *et al.* [79] evaluated the association between tibia lead level and scores on a battery of neuropsychological tests (N = 943–1,140 for the baseline and two follow-up assessments). Previous cross-sectional analyses of these data had revealed relationships between tibia lead levels and test scores [80]. In adjusted analyses, higher tibia lead level was significantly associated with greater decline over time in eye-hand coordination. Tibia lead level-associated deficits were also found in executive functioning, verbal memory, and learning. In the NAS cohort, bone lead level measured 3.5 years earlier, but not concurrent blood or bone lead levels, were associated with scores on tests of response speed, visuospatial skills, and visuomotor skills [81,82].

van Wijngaarden *et al.* [83] used NHANES data (N = 2,299–7,277, depending on the test score) to evaluate, in adults 60 years and older, the association between concurrent blood lead level and two endpoints: self-reported functional limitation due to memory impairment or confusion, and score on the Digit Symbol Substitution Test. Adjusting for age, sex, race, poverty-income ratio, education, and self-reported general health status, no significant associations were found.

Most studies of lead and adult cognitive function have involved only males. Weuve *et al.* [84] assessed lead biomarkers (tibia, patella, blood) in 587 women 47–74 years of age drawn from the Nurses’ Health Study. Mean blood lead level at baseline was 2.9 μg/dL (SD 1.9). All three biomarkers were inversely associated with women’s scores on neurocognitive tests administered five years later.

Several variables have been evaluated as effect modifiers of the association between lead exposure and adult cognition. Among pairs of workers matched in terms of lifetime weighted blood lead level, Bleeker *et al.* [85] found that the inverse association between blood lead level and test scores was more pronounced, at least in certain domains, among the pairs that had low “cognitive reserve” (operationalized as poorer reading achievement), suggesting that greater cognitive reserve protects against lead-associated cognitive impairment. Analyses of both the Baltimore Memory Study and the NAS suggest that greater levels of stress, either self-reported [86] or operationalized as the level of psychosocial hazards in the neighborhood of residence [87], increase an individual’s vulnerability to lead. Finally, several genetic polymorphisms have been investigated. Stewart *et al.* [88] reported that the slope of the inverse association between tibia lead level and cognitive test score was steeper among workers carrying at least one ɛ4 allele of the apolipoprotein E gene. Using NHANES III data, Krieg *et al.* [89,90] reported that modification of the association between lead and cognition by both vitamin D receptor genotypes and ALAD genotypes was complex and differed depending on the age stratum (12–16 years, 20–59 years, >60 years). Chia *et al.* [91,92] and Gao *et al.* [93] suggested that workers carrying the ALAD2 allele are, to some extent, protected against lead neurotoxicity, but in the NAS, men with the ALAD2 allele showed a stronger inverse association between blood lead level and scores on the Mini-Mental Status Examination [94] and a spatial copying test [95]. In the same cohort, however, carriers of the ALAD1 allele were at greater risk of lead-associated changes in mood [96].

Recent studies in rodents and non-human primates suggest a mechanism by which developmental exposure to lead might be a risk factor for neurodegenerative disease in adulthood. Animals exposed to lead only in early life show elevations of beta-amyloid protein precursor (APP) mRNA, APP, and its amyloidogenic product, Abeta, in old age [97]. In monkeys, Abeta staining and amyloid plaques accumulate most striking in the frontal cortex [98]. In addition, DNA methylation is decreased and oxidative damage to DNA increased, suggesting that an epigenetic process might underlie these delayed effects. In mice, maternal lead exposure (*i.e.*, exposure of pups prenatally through weaning) is associated with increased tau phosphorylation and Abeta in pup hippocampus as well as learning deficits on a water maze [99]. *In vitro* studies suggest that the accumulation of Abeta results both from the over-expression of APP and the suppression of the expression of neprilysin, a catabolic peptidase involved in Abeta degredation [100]. No human data are available, although it has been proposed that lead, by this mechanism, might contribute to neurodegenerative disorders such as Alzheimer’s Disease [101].

### 7.6. Adult Psychiatric Status

Opler *et al.* [102,103] followed up, in adulthood, children who had been enrolled in the Childhood Health and Development Study (Oakland, California, USA) and the New England cohort of the National Collaborative Perinatal Project. Cases of schizophrenia spectrum disorder were identified and archived serum samples from pregnancy were analysed for amino levulinic acid, which accumulates when ALAD is inhibited by lead. Based on the relationship between ALA and blood lead level, cases and controls were stratified into groups with a fetal blood lead level estimated to be ≥15 μg/dL or <l5 μg/dL. In pooled analyses of the two cohorts, adjusting for maternal age at delivery and maternal education, the OR associated with an estimated blood lead level ≥15 μg/dL was 1.92 (95% CI 1.05–3.87).

In NHANES 1999–2004, 1,987 20- to 39-year-olds were administered a DSM-IV-based Composite International Diagnostic Interview. Individuals with a current blood lead level in the highest quintile (>2.11 μg/dL; 13 with a level >10 μg/dL), compared with those in the lowest quintile (<0.7 μg/dL), had 2.3 (95% CI 1.1–4.8) times the risk of meeting diagnostic criteria for a major depressive disorder and 4.9 (95% CI 1.3–18.5) times the risk for meeting criteria for panic disorder, adjusting for sex, age, race/ethnicity, education and poverty to income ratio [104]. No association was observed for generalized anxiety disorder. A similar study, however, involving 4,159 individuals 20 years or older in a later round of NHANES (2005–2006) did not find consistent evidence supporting an association between concurrent blood lead level and depression, as assessed by the Patient Health Questionnaire [105].

### 7.7. Brain Imaging

Several recent papers have reported the results of applying various brain imaging modalities to lead workers or, less commonly, to samples drawn from the general population. White matter appears to be particularly vulnerable to injury as a result of lead exposure. Stewart *et al.* [106] found that greater tibia lead level was significantly associated with grade of white matter lesion in 536 former organolead workers. For each μg/g increase in lead concentration, the adjusted OR associated with having a lesion of grade 5+ was 1.04 (95% CI 1.02–1.06, P = 0.004). Because the workers were all at least 15 years removed from occupational exposure, these changes likely represent progressive or persistent structural lesions. However, Schwartz *et al.* [107] did not find an association between former workers’ cumulative lead dose and additional changes in brain volumes or white matter lesion scores over a 5-year follow-up period.

Hsieh *et al.* [108] used diffusion-tensor imaging to compare the integrity of white matter in lead workers (n = 19) to that in age-and sex-matched community controls (n = 18). The mean blood lead level of the workers was 11.5 μg/dL (SD 1.5), compared with 3.2 (SD 1.2) in the controls. Tibia and patella lead levels were also measured. The fractional anisotropy (FA) values of the workers and controls differed significantly bilaterally in parietal, occipital and temporal white matter (all P < 0.05). Moreover, FA values in these regions were significantly associated with the three lead biomarkers. The FA values for the genu and splenium of the corpus callosum did not differ between workers and controls, nor did mean diffusion values in any of the regions measured. These findings suggest that white matter is injured, as reduced FA is considered to reflect axonal damage (fibre orientation and organization) and demyelination. This hypothesis is supported by the results of a study of workers at a primary lead smelter, with blood lead levels that averaged 29 μg/dL (range 16–42) [109]. Damage to white matter, presenting as hyperintensities on T2-weighted magnetic resonance imaging, mediated, at least in part, the inverse association between lead and motor performance.

Grey matter volume in the adult brain is also associated with past lead exposure, as higher tibia lead levels are associated with reduced total brain volume, total grey matter and volumes in several specific regions, including frontal, the cingulate gyrus and the insula [106]. These analyses were adjusted for age, education, height, and apolipoprotein ɛ4 status.

Mounting evidence from the Cincinnati Prospective Lead Study suggests that blood lead levels in childhood predict brain structure and function in young adulthood. Cecil *et al.* [110] and Brubaker *et al.* [111] reported on structural and volumetric imaging studies in young adulthood (mean age 21 years, SD 1.5) of individuals in this cohort. Significant inverse linear associations were found between annual mean blood lead level measured between 3 and 6 years of age and grey matter volume, with the magnitude of volume loss increasing with age. The associations were most striking in frontal regions, particularly the anterior cingulate cortex and ventrolateral prefrontal cortex. Associations were stronger for males than females. In diffusion-tensor imaging studies of this cohort (N = 91), reduced FA and axial diffusivity were found throughout the white matter, as well as changes in the genu, body and splenium of the corpus callosum [112]. Together, these changes suggest lead-related changes in myelination and in axonal integrity.

Lead-related changes in brain function have also been found in this cohort of young adults. Using proton magnetic resonance spectroscopy to measure levels of brain metabolites *in vivo* (e.g., N-acetyl aspartate*,* creatine and phosphocreatine, glycerolphosphocholine and phosphocholines, a composite of glutamate and glutamine), Cecil *et al.* [113] found that blood lead level in childhood predicted reduced levels of metabolites in several regions of grey matter (left basal ganglia, left cerebellar hemisphere, cerebellar vermis) and two regions of white-matter (left frontal, left parietal). Finally, using functional magnetic resonance imaging (n = 42), Yuan *et al.* [114] found significant lead-associated changes in activation patterns in the left frontal cortex and left middle temporal gyrus on a verb generation task.

Changes in brain volume might mediate lead-associated changes observed in adults’ cognitive function. Among former organolead workers, larger volumes in different brain regions were associated with better scores on tests of visuoconstruction, processing speed, visual memory, executive functioning and eye-hand coordination [115]. For the three domains for which test scores were significantly associated with peak tibia lead level (visuo-construction, eye-hand coordination, executive functioning), volumetric mediation was found in that the effect sizes for tibia lead were reduced when volumes of these regions were included as covariates in regression models [116].

### 7.8. Children’s Cognition and Behavior

#### 7.8.1. IQ and Neuropsychological Function

To achieve a more powerful analysis of the quantitative characteristics of the dose-response relationship between children’s blood lead levels and their IQ scores, particularly at blood lead levels <10 μg/dL, the data from seven prospective cohort studies were pooled (N = 1,333) [117]. These studies were conducted in the USA (Boston, Rochester, Cincinnati, Cleveland), Mexico City, Kosovo and Port Piri (South Australia). Four indices of lead exposure history were compared in terms of their relationships to IQ (age 5–10 years): concurrent blood lead level (the level closest in time to the IQ test), maximum blood lead level prior to the IQ test, average lifetime blood lead level (mean level between 6 months of age and IQ measurement) and early childhood blood lead level (mean level from 6 to 24 months of age). Adjustments were made for 10 covariates: HOME Inventory (a measure of the home environment and parental practices and attitudes), sex, birth weight, birth order, maternal education, maternal IQ, maternal age, marital status, prenatal smoking and prenatal alcohol use. A variety of functional forms for the relationship between the different blood lead indices and IQ were compared in terms of their relative fit to the data. A log-linear model for concurrent blood lead level provided the best fit, and suggested a decline of 6.9 (95% CI 4.2–9.4) IQ points over the range of 2.4–30 μg/dL (the upper and lower 5th percentiles of the distribution). Moreover, a restricted spline model, which does not impose a shape on the dose-response relationship, suggested that the steepest decline in IQ occurred at blood lead levels <10 μg/dL. A decrement of 3.9 points (95% CI 2.4–5.3) was associated with an increase from 2.4 to 10 μg/dL; a decrement of 1.9 points (95% CI 1.2–2.6) with an increase from 10 to 20 μg/dL; and a decrement of 1.1 points (95% CI 0.7–1.5) with an increase from 20 to 30 μg/dL. Piecewise linear models were also fit to specific ranges of blood lead levels, defined a priori. Among children for whom the maximum lifetime blood lead level was <7.5 μg/dL (n = 103), the regression coefficient for concurrent blood lead level was −2.94 (95% CI −5.16 to −0.71), compared with a regression coefficient of −0.16 (95% CI −2.4 to −0.08) for children with a maximum blood lead level ≥7.5 μg/dL (P = 0.015). The results did not depend unduly on the data of any one study, as the coefficient for concurrent blood lead level changed only by −2.6 to +8.6% when the data from any one of the seven studies were excluded.

A similar, supra-linear, relationship has been reported in other, independent studies since the publication of this pooled analysis (e.g., [118,119]). Tellez-Rojo *et al.* [119] evaluated blood lead level and neurodevelopment at 12 and 24 months in 294 children in a prospective study in Mexico City. Analyses were restricted to children whose blood lead levels were <10 μg/dL at both 12 and 24 months. Adjusting for covariates, blood lead level at 24 months was significantly associated, inversely, with both mental and motor development scores at 24 months, whereas blood lead level at 12 months was inversely associated with the motor development score at 24 months, but not with concurrent mental or motor development. For both mental and motor development scores at 24 months, the coefficients for concurrent blood lead level were significantly larger among children with levels <10 μg/dL than among children with blood level levels ≥10 μg/dL.

Numerous other studies have reported adverse outcomes in children at blood lead levels <10 μg/dL. In a cross-sectional study of 534 6- to 10-year-old children, adjusting for age, race, socioeconomic status and caregiver IQ, children with blood lead levels of 5–10 μg/dL, compared to children with a level of 1–2 μg/dL, had a 5 point deficit in IQ, a 7.8 point deficit in reading, a 6.9 point deficit in mathematics, as well as deficits in spatial attention and executive functions [120].

In a cohort of 246 7.5-year-old African American children (mean blood lead level of 5.4 μg/dL, range 1–25 μg/dL), Chiodo *et al.* [121] found significant covariate-adjusted inverse associations between blood lead level and scores on a variety of neuropsychological tests. Analyses stratifying by blood lead level (≤5, 5–10, >10 μg/dL) suggested that the associations became significant when blood lead level exceeded 5 μg/dL.

Solon *et al.* [122] reported on population-based stratified random sample of 877 children age 6 months to 5 years in the Philippines, in whom the mean blood lead level was 7.1 μg/dL. Adjusting for covariates, each μg/dL increase in blood lead level was associated with a 3.3 point decline in neurodevelopmental score in younger children and a 2.5 point decline in older children. Nutritional deficiencies (folate, iron), were effect modifiers, exacerbating the lead-associated decline in scores.

In a cohort of 261 8- to 11-year-old children, blood lead level (mean 1.7 μg/dL, range 0.42–4.91) was inversely associated with IQ score (coefficient −0.18, P = 0.003), adjusting for age, sex, maternal education, paternal education, income, maternal smoking during pregnancy, exposure to second-hand smoke after birth, birth weight, maternal age at birth and blood manganese level [123]. An additive interaction was observed between blood lead and manganese levels, with a steeper lead-associated IQ decline observed among children with a manganese level greater than the median value (14 μg/l). A similar interaction was observed by Claus Henn *et al.* [124]. The initial reports of a supra-linear relationship between blood lead level and IQ at blood lead levels <10 μg/dL compared with ≥10 μg/dL generated concern that this might be merely a statistical artefact. Bowers & Beck [125] argued, for instance, that “the dose-response curve between an environmental measure that has log-normal distribution and any cognitive score that is normally distributed will by necessity have a non-linear slope” (p. 523). Several contested this statement [126–133], but the issue is moot. In the international pooled analysis, piecewise linear models fit to different ranges of blood lead (e.g., <7.5 μg/dL, <10 μg/dL) showed that the linear slopes were significantly steeper in the lower than in the higher ranges, and that linear fits were satisfactory within these more restricted ranges. Moreover, for each of the individual seven studies, a linear model provided the best fit across the blood lead range represented in the study cohort. As noted a decade earlier [134], the inverse slopes tended to be larger in cohorts with lower mean blood lead levels than in cohorts with higher blood lead levels. It is not surprising, then, that when the studies were combined in a pooled analysis, the functional form that provided the best fit over the broader blood lead range covered by the combined studies was non-linear.

The potential import of the lead-associated reductions in IQ (and other cognitive functions) are suggested by studies conducted by Miranda *et al.* [135], who linked state-wide databases in North Carolina, USA providing children’s blood lead levels and their scores on an end-of-grade (4th grade) reading test. In the first study (N = 8,603), children with a higher blood lead level were at significantly increased risk of failing the reading test, with the association evident at levels as low as 2 μg/dL. In a second study [136], blood lead screening levels at age 9–36 months and end-of-4th grade reading score were available for 56,678 children. Children with a blood lead level of one μg/dL were the referent group, and dummy variables were created to represent groups of children with each integer value of blood lead. The coefficients for all blood lead categories were inverse and significant. For children with a blood lead level of 2 μg/dL, the coefficient was −0.30 (95% CI −0.58 to −0.01); for children with a blood lead level of 5 μg/dL, it was −0.80 (95% CI −1.08 to −0.51); and for children with a blood lead level greater than 10 μg/dL, it was −1.75 (95% CI −2.09 to −1.41). Quantile regression analyses suggested that higher blood lead levels had a disproportionate impact on children who, for reasons other than lead, were at risk of reading difficulties. Specifically, the adverse impact of lead exposure on reading was greatest among children whose scores, for other reasons, placed them in the lower tail of the reading distribution. In other words, the impact of lead was magnified in the presence of other developmental risk factors.

The potential long-term implications of early-life lead exposure are suggested by the results of a follow-up, at approximately age 30 years, of children who had been enrolled, at birth, in the Boston Prospective Study [137]. Although only 43 of the original participants could be located and assessed, IQ in adulthood was significantly related to mean blood lead level in middle childhood (mean at age 4: 6.7 ± 3.6 μg/dL; mean at age 10 years: 3.0 ± 2.7 μg/dL).

#### 7.8.2. Attention Deficit Hyperactivity Disorder (ADHD)

Older studies using blood or tooth lead levels as the primary exposure biomarker consistently identified dose-related increases in behavioral outcomes such as inattention, distractibility and hyperactivity [138–141]. The exposures of the children enrolled in these studies were considerably higher than the exposures of contemporary children, however, and the outcomes were generally based on teacher or parent reports rather than on formal diagnostic evaluations. Recent studies address these limitations.

In NHANES 1999–2002, parents reported whether they had ever been told by a health professional that their 6- to 16-year-old child met criteria for ADHD and whether the child was taking a medication for ADHD. Braun *et al.* [142] found that the children in the fifth blood lead quintile (>2 μg/dL) were 4.1 (95% CI 1.2–14.0) times more likely than children in the first quintile (<0.8 μg/dL) to have ADHD. Children in the second, third, and fourth quintiles were 1.1 (95% CI 0.4–3.4), 2.1 (95% CI 0.7–6.8), and 2.7 (95% CI 0.9–8.4) times more likely to have ADHD, respectively. Adjustments were made for age, sex, prenatal and postnatal exposure to environmental tobacco smoke, preschool or child-care attendance, health insurance coverage and serum ferritin level.

Froehlich *et al.* [143] analysed data for 2,588 8–15 year olds in NHANES 2001–2004, but in these analyses, the diagnosis of ADHD was based on the Diagnostic Interview Schedule for Children, a structured interview based on DSM-IV. Children with a current blood lead level in the upper tertile (>1.3 μg/dL) were 2.3 (95% CI 1.5–3.8) times more likely than children in the lowest tertile to have ADHD. Children with both prenatal exposure to tobacco and a current blood lead level in the upper tertile were at particular risk (adjusted OR 8.1, 95% CI 3.5–18.7).

In a study of 1,778 children in whom blood lead levels ranged from 0.1 to 10.1 μg/dL (geometric mean 1.8 μg/dL) and for whom parents completed on the Connors’ scale for ADHD, the risk of ADHD, defined as a score exceeding the cut-off derived for children from the Republic of Korea, increased linearly with increasing blood lead level [144]. These analyses were adjusted for age, sex, income, place of residence, parental history of neuropsychiatric disease (but not specifically ADHD), and blood mercury level. Children a blood lead level >3.5 μg/dL were 1.96 (95% CI 0.76–5.11) times more likely than children with a blood lead level <1 μg/dL to have ADHD, and the P for trend across blood lead categories was 0.07. Blood lead level was positively associated with the number of ADHD symptoms endorsed (P < 0.001), although it appears that this association was largely attributable to children with a blood lead level >5 μg/dL.

In a study of children 4–12 years old, 630 cases who met DSM-IV diagnostic criteria were matched to 630 controls on age, sex and socioeconomic status [145]. In a conditional logistic regression analysis in which children with a blood lead level <5 μg/dL were the referent group and adjustments were made for household composition, birth weight, family history of ADHD, pregnancy, labour and delivery complications, medical history, maternal and paternal age, maternal and paternal education, and use of alcohol and cigarettes during pregnancy, risk of ADHD was 5.2 (P < 0.01) among children with a blood lead level of 5–10 μg/dL were 5.2 times more likely to have ADHD, while children with a blood lead level >10 μg/dL were 7.2 times as likely to have ADHD.

Nigg *et al.* [146] used a multistage screening and verification process to confirm a diagnosis of ADHD using DSM-IV criteria and to rule out co-morbidities in a sample of 150 8- to 17-year-old children (97 cases and 53 controls). Blood lead level ranged from 0.40 to 3.47 μg/dL (mean 1.03). Blood lead level was significantly related to the ADHD symptom count for total symptoms and for hyperactivity-impulsivity counts (P < 0.05). Adjusting for income and sex, children with ADHD combined subtype had a higher blood lead level than did controls (P < 0.04). In an additional study of 236 6- to 17-year-olds, 108 of whom met diagnostic criteria for ADHD, adjusting for IQ and parental smoking, blood lead level (mean 0.73 μg/dL, maximum 2.2) was associated with risk of ADHD combined subtype, but not the inattentive subtype [147]

In a study conducted in Romania (N = 83, 8–12 years old), Nicolescu *et al.* [148] found that blood lead level (median of 3.7 μg/dL), but not blood mercury (median of 0.1 μg/dL) or aluminum (median of 3.3 μg/dL) levels, were significantly associated with core features of ADHD, as assessed by computer tasks (German KITAP battery) as well as by parent and teacher ratings. The results were similar when analyses were restricted to children a with blood lead level <10 μg/dL.

#### 7.8.3. Violence and Aggression

In an early case series, Byers and Lord [149] noted that lead-poisoned children exhibited explosive tempers and poor impulse control, but this observation was not followed-up and explored more deeply for several decades. Denno [150] assembled retrospective evidence, from the Collaborative Perinatal Study, that childhood lead poisoning is a surprisingly strong risk factor for juvenile crime. Systematic study of the association between lower levels of lead exposure and aggression began in earnest, however, with Needleman *et al.* [151]. In this study, parent- and teacher-rated scores in the range of clinical concern on the aggression scale of the Child Behavior Checklist were more common among children with higher bone lead levels. Needleman *et al.* [152] followed this study up with a case-control study of 216 adjudicated delinquents. Adjusting for race, parent education, parent occupation, family size, presence of two biological parents and presence of two parental figures in the home, the OR associated with having an elevated bone lead level was 2.0 (95% CI 1.1–31.0) among delinquent boys and 7.8 (95% CI 1.7–35.0) among delinquent girls.

Studies using an ecological design have reported significant associations between air lead concentrations and homicide rate [153] and property and violent crime rates [154] and between lead production and the murder rates in the United States [155]. In a subsequent set of analyses, using aggregated data from Australia, Canada, Finland, France, Germany, Great Britain, Italy, New Zealand and the USA, Nevin [156] examined the association between preschool blood lead level and different types of crime. Relative fits were compared for models incorporating lags of various durations between putative exposure and the occurrence of the outcomes. The lags that provided the best fits were consistent with the known peak offending ages for various offences (e.g., burglary *vs.* homicide). In an ecological study such as this one, it is not possible to draw inferences because of limited ability to adjust for potential confounders.

This limitation has been addressed, at least in part, in several recent cohort studies. Braun *et al.* [157] used data for 2,619 children 8–15 years of age in NHANES 1999–2002 to evaluate the association between concurrent blood lead level and the diagnosis of conduct disorder. By parent report, 68 children met DSM-IV criteria. Adjusting for age, maternal age, sex, race, prenatal tobacco exposure and serum cotinine, and using children in the lowest quartile of blood lead as the referent group (0.8–1.0 μg/dL), the OR was significant for quartile 3 (1.1–1.4 μg/dL) (OR = 12.4, 95% CI 2.4–64.6) and quartile 4 (1.5–10 μg/dL) (OR = 8.6, 95% CI 1.9–40.0).

In a cross-sectional study of 173 14- to 18-year-olds in Brazil, surface dental enamel lead level was associated, after adjusting for familial and sociodemographic confounders, with the risk of clinically significant elevation of parent-reported rule-breaking behaviour on the Child Behavior Check List (OR = 3.72, 95% CI 0.99–14.04) [158]. Enamel lead level was not associated with children’s self-report of delinquent behaviours, however.

Three prospective studies of environmental lead exposure and criminal activities have been reported. In the Christchurch Health and Development Study, a birth cohort of 1,265 children, dentine lead level in a deciduous tooth, measured at 6–9 years of age, was related, in a dose-dependent manner, to the number of violent/property convictions and self-reported violent/property offenses committed between the ages of 14 and 21 [159]. The effect sizes for both outcomes were reduced by adjustment for sociodemographic variables and aspects of family functioning, but remained significant (P < 0.005 for convictions and 0.047 for self-reported offences). Additional analyses suggested that educational underachievement (leaving school without qualifications, low grade point average) mediated these associations, at least in part.

Among 488 children in the Avon Longitudinal Study of Parents and Children (UK), higher blood lead level at 30 months of age was significantly associated with greater antisocial behaviors at age 7–8, as reported by an adult [160]. The association was strongest among children with a blood lead level ≥10 μg/dL.

The strongest epidemiological evidence germane to the hypothesis that early life lead exposure increases the propensity to criminality has come from the Cincinnati Prospective Study, which has followed children from pregnancy into young adulthood. It had been reported previously that adolescents in this cohort with higher blood lead levels in early childhood had engaged in more delinquent acts, but these outcome data were based on adolescents’ self-reports [161]. In a follow-up study conducted when the participants were 19–24 years olds (N = 250), county records of arrests were collected [162]. Detailed blood lead histories were available up to the age of 6.5 years. The median prenatal blood lead level (first or second trimester) was 7.8 μg/dL (range 2.9–16.0); the median early childhood average blood lead level was 12.3 μg/dL (range 6.0–26.3); and the median blood lead level at 6.5 years was 6.8 μg/dL (range 3.4–18.3). In analyses of total arrests after age 18, adjusting for maternal IQ, sex, socioeconomic status and maternal education, the rate ratios associated with each 5 μg/dL increase were significant for prenatal blood lead level (1.4, 95% CI 1.07–1.85) and 6-year blood lead level (1.3, 95% CI 1.0–1.6). In analyses of arrests for violent offences, the adjusted rate ratios were significant for average childhood blood lead level (1.3, 95% CI 1.0–1.6) and for 6-year blood lead level (1.5, 95% CI 1.1–1.9). Among the strengths of this study are the prospective collection of data on exposure and confounders, and the use of administrative records rather than self-report as the source of data on outcome.

In a meta-analysis, Marcus *et al.* [163] combined the results of 19 studies that evaluated the associations between blood, bone, tooth, or hair lead levels and delinquency or criminality, conduct problems, and aggressive or oppositional behavior, all of which were considered to reflect conduct problems. The average correlation between the lead biomarker and behaviour problems was 0.19 (0.15 excluding the 3 studies that relied on hair lead, a biomarker of questionable validity). This is similar in magnitude to the correlation typically observed in children between blood lead level and IQ [164].

The biological plausibility of the hypothesis that elevated lead exposure is causally associated with aggression is supported by studies in experimental models, including cats [165], primates [166], and hamsters [167]. While these animal studies suggest a direct association, the propensity to increased aggression and violence in children could also reflect indirect pathways of influence. For example, individuals with lead-related IQ loss, ADHD, impairments of executive function, and poor impulse control might be at increased risk of manifesting such behaviors.

## 8. Delayed Sexual Maturation

Higher blood lead level has been associated with delayed sexual maturation in cross-sectional studies. Among 2,186 8–18 year old girls in NHANES II, breast and pubic hair development (Tanner staging) as well as age at menarche were significantly delayed in African-American and Mexican American girls with a blood lead level ≥3 μg/dL, compared with girls with a blood lead level of 1 μg/dL [168]. Each 1 μg/dL increase in blood lead level was associated with a delay of 2.1–6.0 months in progressing from one Tanner stage to the next. The delay in age at menarche among girls with a blood lead level ≥3 μg/dL was 3.6 months. These indices of maturation were not significantly delayed in white females, however.

Wu *et al.* [169] studied girls 10–16 years of age (NHANES III). Data on age at menarche were available for 1,235 girls, and data on Tanner stage 2 pubic hair and breast development (determined by a physician) were available for 1,706 girls. Blood lead level was categorized (0.7–2.0 μg/dL, 2.1–4.9 μg/dL, 5.0–21.7 μg/dL). Blood lead level was inversely related to both pubic hair development and age at menarche, but not breast development, adjusting for race/ethnicity, age, family size, residence in a metropolitan area, poverty-to-income ratio and body mass index. In the three blood lead groups, 60.0%, 51.2% and 44.4% of 10-year-olds, respectively, had reached Tanner stage 2 for pubic hair, and 68.0%, 44.3% and 38.5% of 12-year-olds, respectively, had reached menarche.

In a study of 138 10- to 17-year-old girls from the Akwesasne Mohawk Nation in the USA, Denham *et al.* [170] found that, among those with a blood lead level above the median (1.2 μg/dL), menarche was reached 10.5 months later than it was among girls with a blood lead level below the median, adjusting for age, socioeconomic status and other pollutants (dichlorodiphenyldichloroethylene, hexachlorobenzene, mirex, mercury).

Sexual maturation in boys (physician-assessed testicular volume and genitalia stage) was evaluated in 489 8- to 9-year-old boys in Chapaevsk, Russian Federation [171]. The median blood lead level was 3 μg/dL (interquartile range 2–5). In analyses adjusting for birth weight, gestational age and age at examination, boys with a blood lead level ≥5 μg/dL had were less likely than boys with a blood lead level <5 μg/dL to have reached genitalia stage 2 (OR = 0.6, 95% CI 0.3–0.95, P = 0.03). This cohort was followed up (N = 481) several years later [172], when more boys had reached puberty. Adjusting for baseline body mass index and height, boys with a baseline blood lead level ≥5 μg/dL had a reduced likelihood of pubertal onset based on testicular volume (HR = 0.73, 95% CI 0.55–0.97), genitalia staging (HR = 0.76, 95% CI 0.59–0.98) and pubic hair staging (HR = 0.69, 95% CI 0.44–1.07). The effect sizes corresponded to onset delays of 6–8 months.

## 9. Dental Health

In adults, greater lead exposure has been associated with risk of tooth loss. In the NAS cohort, men in the highest tertile of tibia lead level were 3.0 (95% CI 1.6–5.8) times more likely than men in the lowest tertile to have more than nine missing teeth. Men in the highest tertile of patella lead level were 2.4 times as likely (95% CI 1.3–4.5) [173] to have more than nine missing teeth. Among 4,899 men and women 20–56 years of age in NHANES III (1988–1994), the adjusted prevalence of periodontitis (presence of more than 20% of mesial sites with greater than or equal to 4 mm of attachment loss) was greater among participants with a blood lead level >7 μg/dL (men: prevalence ratio 1.7, 95% CI 1.0–2.9; women: prevalence ratio 3.8, 95% CI 1.7–8.7) than among men and women with a blood lead level <3 μg/dL [174]. Similar findings were reported in smaller studies [175,176].

In children, lead exposure has been reported to be a risk factor for dental caries, but the evidence is mixed and lacks consistency across studies in terms of the patterns of associations. Among 24,901 participants ages 2 years and older in NHANES III (oldest age stratum was ≥12 years), a higher blood lead level was significantly associated with the number of delayed, filled, and missing surfaces in both deciduous and permanent teeth, adjusting for age, race, poverty-to-income ratio, cigarette exposure, sex, region, parent education, carbohydrate intake, dietary calcium intake and dental care [177]. Among 5 to 17 year old children, a 5 μg/dL increase in blood lead level was associated with a 1.8-fold (95% CI 1.3–2.5) increase in the risk of caries. Children with blood lead levels in the upper tertile of the distribution (>3 μg/dL) were 1.7 (95% CI 1.1–2.5) times as likely to have caries.

In 543 6- to 10-year-old children enrolled in the New England Children’s Amalgam Trial [178], a positive association between caries and blood lead level (mean 2.3, SD 1.7 μg/dL) was observed among the children recruited from an urban area (P = 0.005), adjusting for age, sex, family income, ethnicity, maternal education, maternal smoking, dental hygiene habits (frequency of brushing, firmness of brush) and gum chewing. This association was somewhat stronger in deciduous than in permanent teeth. No association was observed in children recruited from a rural area, however. The ranges of both blood lead level and the number of carious tooth surfaces were greater in the urban than in the rural subgroup, which might have made it easier to detect an association. Alternatively, the possibility of residual confounding or the influence of effect modifying factors whose distributions differed between regions cannot be dismissed.

In analyses of 507 8- to 12-year-old children participating in another study of dental amalgam, Martin *et al.* [179] reported that blood lead level (mean 4.6 μg/dL, SD 2.4) was significantly associated with number of carious surfaces, but only among males, and only in primary teeth (adjusting for age, race, IQ and scores on tests of attention, memory and visuomotor function). In contrast, in a study of 292 6- to 11-year-old children in Thailand whose mean blood lead level was 7.2 (SD 1.5) μg/dL, a doubling of blood lead level was associated with a 2.4 times (95% CI 1.4–4.2) increase in the risk of having more than 5 decayed/filled surfaces in deciduous teeth, but not in primary teeth [180]. In a retrospective study of blood lead level and the number of decayed, filled and missing surfaces in second and fifth graders, children with a mean blood lead level ≥10 μg/dL between 18 and 37 months of age were not at increased risk of having a greater number of delayed, filled, and missing surfaces [181].

## 10. Conclusions

Although IQ deficit in children is typically chosen as the critical endpoint for the purpose of risk assessment, recent studies, primarily conducted in developed countries, show that many aspects of health are impaired at exposure levels prevalent in the general (*i.e.*, non-occupationally exposed) population and at levels similar to those associated with IQ loss in children, including all-cause mortality, renal function, cardiovascular function, psychiatric status, sexual maturation, and dental health.

Recent risk assessments have concluded that a “safe level” of lead exposure has not been identified. For example, in 2010, the FAO/WHO Joint Expert Committee on Food Additives (JECFA) argued that dose-response analyses do not provide any indication of a threshold for critical adverse effects (IQ loss in children, increased blood pressure in adults) and, as a result, withdrew its Provisional Tolerable Weekly Intake (PTWI) of 25 μg/kg body weight [182]. Furthermore, it concluded that the evidence precluded the identification of a new PTWI that would be health protective. Similarly, in 2010, the European Food Safety Authority’s Panel on Contaminants in the Food Chain also concluded that the JECFA’s PTWI is no longer appropriate [183]. In the U.S., the Advisory Committee on Childhood Lead Poisoning is currently deliberating on whether to revise or eliminate its “level of concern.”

A disproportionate share of the morbidity associated with lead exposure is borne by developing countries. The World Health Organization noted that, in 2000, approximately 10% of children had a blood lead level of 20 μg/dL or higher, but that 99% of these children lived in developing countries and that lead exposure accounted for nearly 1% of the global burden of disease [184]. Lead’s share of the global burden is likely to have decreased in the last decade due to control measures such as elimination of use as a gasoline additive, but episodes of severe lead poisoning continue to be reported. For example, in 2010, the Nigerian government sought international assistance in addressing an outbreak in several villages, resulting from local processing of gold ore rich in lead, in which 118 of 463 children under 5 years of age died and 97% of surviving children had a blood lead level ≥45 μg/dL (ranging up to 445) [185]. Although substantial progress has been made in reducing the human suffering that can be attributed to lead, the “problem” is far from solved.
